# Lipid Raft in Cardiac Health and Disease

**DOI:** 10.2174/157340309788166660

**Published:** 2009-05

**Authors:** Manika Das, Dipak K Das

**Affiliations:** Cardiovascular Research Center, University of Connecticut School of Medicine, Farmington, CT 06030-110, USA

## Abstract

Lipid rafts are sphingolipid and cholesterol rich micro-domains of the plasma membrane that coordinate and regulate varieties of signaling processes. Lipid rafts are also present in cardiac myocytes and are enriched in signaling molecules and ion channel regulatory proteins. Lipid rafts are receiving increasing attention as cellular organelles contributing to the pathogenesis of several structural and functional processes including cardiac hypertrophy and heart failure. At present, very little is known about the role of lipid rafts in cardiac function and dysfunction. This review will discuss the possible role of lipid rafts in cardiac health and disease.

## INTRODUCTION

The traditional model of plasma membrane as a homogeneous fluid lipid bilayer, as demonstrated by Singer and Nicholson (1972), has been extensively modified in recent years, and it has become increasingly clear that plasma membrane consists of numerous lipids that constitute much more complex structure than previously thought. However, work over last decade has provided evidence that the plasma membrane is not a random ocean of lipids; rather, there is structure within this sea of lipids that in turn imposes organization on the distribution of proteins in the bilayer. The lipid “structures” within the membrane ocean are called lipid rafts [1]. The fatty-acid chains of lipids within the raft tend to be extended and so more tightly packed, creating domains with higher order. It is therefore thought that rafts exist in a separate ordered phase that floats in a sea of poorly ordered lipids.

Lipid rafts are sphingolipid and cholesterol-rich-domains of the plasma membrane which contain a variety of signaling and transport proteins. Different subtypes of lipid rafts can be distinguished according to their protein and lipid composition. Caveolae, a subset of lipid rafts, are flask-like invaginations of plasma membrane that contains proteins of caveolin family (Caveolin-1, caveolin-2 and caveolin-3) [1]. The presence within lipid rafts of a variety of membrane proteins involved in cell signaling has led to the consensus that these lipid domains play an important role in the process of signal transduction [2]. In some cases, preassembled signaling complexes are localized in this lipid raft domains [2].

## LIPID RAFT AND SIGNALING COMPONENTS

A large number of GPCR (G-protein coupled receptor) have been reported to co-localize with lipid raft/ caveolae. In case of Angiotensin I receptor, GPCR-caveolin interaction is important for receptor sorting and delivery to plasma membrane [3]. According to the caveolin signaling hypothesis, caveolae bring downstream effectors in proximity to receptors (e.g., GPCRs) for initiating receptor, tissue and cell-specific signal transduction [4, 5]. These effectors are thought to reside within caveolae by direct interaction with caveolin. Palmitoylation may enhance caveolar localization of proteins [6, 7].

Among the different binding proteins of caveolin, its interaction with eNOS has been most extensively studied [8]. Binding of eNOS with caveolin inhibits enzyme activity [9] and loss of caveolin expression upregulates eNOS activity [10]. Like eNOS, caveolin is also thought to negatively regulate Adenylate Cyclase (AC) activity. Caveolin-1 and caveolin-3, but not caveolin-2 inhibits AC activity and this inhibition is AC isoform specific [11]. Like eNOS, protein kinases (PKA/PKC) can also interact with caveolin-1 and inhibit its activity [12]. The PKC family of enzymes translocate to the cellular compartment in response to the external stimuli [13]. The phosphatidylinositol-3-kinase/protein kinase B (PI3K/PKB, Akt) pathway is another protein kinase system that interacts with caveolin and this interaction may regulate cell survival. For example, caveolin retains Akt in activated form (phosphorylated form) in prostate cancer [14], presumably *via *interaction with caveolin scaffolding domain of caveolin and by inhibition of protein phosphatase 1 and 2A [15]. In muscle, we can also found a linear relationship between the expression of caveolin-3 and activation of PI3K/Akt pathway in the regulation of cell survival [16]. In addition, the phosphorylated form of caveolin is involved in EGF receptor transactivation, which is dependent on Src and Akt phosphorylation and for which caveolin helps integrate this signaling cascade [17].

Receptor tyrosine kinases also have been localized to caveolae [e.g., EGF, NGF, IGF and PGDF] and their downstream effectors MAP kinases, which regulate numerous cellular processes, are also regulated by caveolin [18, 19]. P42/44 MAPK localizes to caveolae and is negatively regulated by interaction with caveolin 1 [20]. Overexpression of caveolin-1 also inhibits the MEK/ERK signaling pathways [21]. Consistent with this action, caveolin-1 and-3 knock out mice showed increased activation of p42/44 MAPK [22]. Ischemia reperfusion showed differential activation of p42/44 ERK and p38MAPK in cavaeolar and noncaveolar fraction, indicating differential regulation of these kinases by caveolin [23]. Certain nonreceptor tyrosine kinases such as members of src family (c-Src, Fyn, lyn) are enriched in caveolae and interactions with caveolin-1 also suppress the kinases activities [24, 25]. Tyrosine phosphorylation of caveolin itself makes phospho caveolin, which acts as a key site of tyrosine kinase signaling [26].

## CAVEOLIN KNOCKOUT AND PHENOTYPE

The most appropriate approach for the study of caveolin is the use of knock out (KO) mice. Caveolin-KO mice (Cav-1,-2, -3) and caveolin 1/3 double KO mice have already been developed. Although they are viable, they are fertile but display numerous phenotypes. Caveolin-1 knockout mice develop progressive cardiac hypertrophy as demonstrated by transthoracic echocardiography (TTE) and magnetic resonance imaging (MRI) [22]. In contrast, caveolin-3 knockout mice develop cardiomyopathy characterized by hypertrophy, vasodilatation and reduced contractility as well [27]. Caveolin-1 and caveolin-3 double knockout mice completely lacking caveolae are deficient in all three caveolin proteins because caveolin-2 is degraded in absence of caveolin-1. The double knockout mice developed severe cardiomyopathic phenotype with cardiac hypertrophy and decreased contractility [28]. Additionally, Cav-1 KO mice exhibited myocardial hypertrophy, pulmonary hypertension and alveolar cell hyper proliferation caused by constitutive activation of p42/44 mitogen activated protein kinase and Akt [29] Interestingly, in Cav-1-reconstituted mice, cardiac hypertrophy and pulmonary hypertension were completely rescued [29]. Again, genetic ablation of Cav-1 leads to a striking biventricular hypertrophy and to a sustained eNOS hyper-activation yielding increased systemic NO levels [30]. Furthermore, a diminished ATP content and reduced level of cyclic AMP in hearts of knockout mice was also reported [30]. Taken together, these results indicate that genetic disruption of caveolin-1 is sufficient to induce severe biventricular hypertrophy with signs of systolic and diastolic heart failure [30].

Apart from its ability to degrade extracellular matrix proteins, matrix metalloproteinase-2 (MMP-2) was recently revealed to have targets and actions within the cardiac myocyte. MMP-2 (gelatinase A) has been localized to the thin and thick myofilaments of the cardiac sarcomere, as well as to the nucleus [31, 32]. The intracellular proteins troponin I and myosin light chain-1 are proteolyzed by MMP-2 in ischemia/reperfusion injury [31, 32]. The tissue inhibitors of metalloproteinase (TIMPs) control MMP activities [33], but other mechanisms of regulation are less well elucidated. In endothelial cells, MMP-2 has been localized to the caveolae [34] yet its function there is unknown. Disruption of caveolae activates MMP-2 in fibrosarcoma cells [35] while Cav-1 overexpression in tumor cells causes decreased MMP-2 activity [36] suggesting that Cav-1 may participate in the regulation of MMP-2. Whether the role of MMP-2 activity in the heart is affected by caveolin still remains unknown. Here we present evidence that MMP-2 localizes with Cav-1 in the mouse heart, and that CSD inhibits MMP-2 activity and that hearts of mice deficient in Cav-1 have increased MMP-2 activity.

Interestingly, Cav-3 KO mice show a number of myopathic changes, consistent with a mild to moderate muscular dystrophy phenotype. However, it remains unknown whether a loss of cav-3 affects the phenotypic behavior of cardiac myocytes *in vivo*. Cav-3 knockout hearts display significant hypertrophy, dilation and reduced fractional shortening as revealed by gated cardiac MRI and transthoracic echocardiography. Histological analysis reveals marked cardiac myocyte hypertrophy, with accompanying cellular infiltrates and progressive interstitial/ peri-vascular fibrosis. It has also demonstrated that p42/44MAPK (ERK1/2) is hyperactivated in heart derived from caveolin-3 knockout mice, which can lead to cardiac hypertrophy [37].

In the endoplasmic reticulum, Cav-3 initiates the biogenesis of caveolae organelles by forming homooligomers and hetero-oligomers with Cav-1 [38]. At the plasmalemma, Cav-3 interact with dystrophin and its associated glycoproteins [39, 40]. Cav-3 and dystrophin competitively bind to the same site of *β*-dystroglycan, suggesting that Cav-3 may regulate the membrane recruitment of dystrophin and the assembly of the dystrophin glycoprotein complex (DGC) [41]. At the cell surface, Cav-3 colocalizes also with signaling molecules such as Gi2*α*, G*β γ*, c-Src, other Src kinases as well as nitric oxide synthases (neuronal and inducible NOS), indicating that muscle caveolae might be involved in the modulation of these signaling processes [42, 43]. In addition, Cav-3 plays a role in the regulation of energy metabolism of muscle cells as it is required for the cell membrane targeting of phosphofructokinase, an enzyme that catalyzes a rate-limiting reaction in glycolysis [44].

*In vitro* studies have shown that Cav-3 plays a critical role in myoblast cell differentiation and survival and in myotube formation [45]. The relevance of Cav-3 in muscle physiology was further confirmed by the findings that mutations in the CAV3 gene result in distinct neuromuscular and cardiac disorders, such as limb girdle muscular dystrophy (LGMD) 1-C, idiopathic persistent elevation of serum creatine kinase (hyperCKemia), inherited rippling muscle disease (RMD), distal myopathy and familial hypertrophic cardiomyopathy (HCM) [46-48].

The *CAV3* gene (OMIM no. 601253) spans 12 kb of genomic DNA on chromosome 3p25 and contains two exons. At present, 20 different point mutations, 2 base-pair deletions and 1 novel splice site mutation have been reported [49]. More recently, four novel *CAV3* mutations have been identified in patients affected by congenital long-QT syndrome (LQTS) in the absence of signs of primary cardiomyopathy, suggesting a possible role for Cav-3 in the regulation of cardiac ion channels [49, 50].

## CAVEOLAE AND CARDIAC ION CHANNELS

Modulation of ion channel activity plays a critical role in regulating cardiovascular function. Recently, it has become apparent that the regulation of channel function is not the only means of controlling excitability, the trafficking and localization of ion channels with signaling molecules also play a significant role. Most cells in the cardiovascular system express multiple channel types (e.g., voltage-gated Na^+^, K^+^ and Ca^2+^ channels) and even multiple isoforms of a particular channel, with each channel uniquely contributing to excitability [51, 52]. Voltage gated Na^+^ channels are responsible for the initial depolarization of the cardiac sarcolemma, to permit the opening of voltage-gated L-type Ca^2+^ channels, resulting in Ca ^2+^ influx and contraction. Membrane repolarization is controlled by K^+^ channels. Therefore, altering the number of channels and/or their function can have significant impact on both resting membrane potential and the cardiac action potential wave form. Defects in either of these processes can have life-threatening implications [51, 52].

In several cell types, including smooth muscle and endothelial cells, mediators of calcium signaling, such as Ca^2+^-ATPase, inositol-triphosphate receptor (IP3R), Ca^2+^ pumps and L-type Ca ^2+^ channels, large conductance Ca^2+^ activated K^+^ channel, calmodulin and transient receptor potential (TRP) channels, localize in cholestetrol-rich membrane domains. Such localization suggest that membrane raft and/or caveolae have a role in calcium handling and Ca^2+^ entry that control excitation-contraction of heart muscle [53-55]. TRP channels, in particular TRPC1, -3 and -4 are enriched in caveolae and caveolin-1 regulates the plasma membrane localization and function of TRP channels [56]. Current evidence indicates that caveolae regulate calcium entry and depletion of cholesterol by methyl-β-cyclodextrin reduces colocalization of caveolin-1 and TRPC1 and redistribution of TRPC1, thus preventing Ca^2+^ influx [57]. Moreover, Na^+^ pump, Na/K-ATPase, contains two caveolin binding motifs and resides in caveolae in a number of cells, including smooth muscle cells and cardiomyocytes, thereby helping to maintain Na^+^ gradient [58]. Voltage gated K^+^ channels are also localized in caveolae and play an important role to maintaining cellular excitability. In fibroblast, the Kv 1.5 subunit colocalizes with caveolin-1, Kv 2.5 localizes with membrane raft and depletion of cholesterol with MβCD redistributes and alters the function of K^+^ channel [59]. These findings imply that alteration of caveolae and/or caveolin by any disease or drug treatments can shift the localization of the channels, thereby altering cellular excitability and functional activity.

## CAVEOLAE AND CARDIOVASCULAR DISEASE

There is a vast literature about the roles of caveolae and caveolin in the regulation of many cellular processes in cultured cells and many investigators considered them as an essential platform of signaling molecules. However, in the past few years, development of animal models and usage of genetically altered mice have been instrumental in deciphering their physiological functions *in vivo*. Transgenic over expression of caveolin-1 or caveolin-3 in mice or targeted disruption of each of the caveolin gene locus in mice (Cav-1, Cav-2 and Cav-3 genes) has provided significant insight into the roles of caveolin and caveolae [60]. The potential role of caveolin in cardiovascular physiology has become apparent by the discovery of cavelin-1 and caveolin-3 KO mice and double knockout mice, which have cardiomyopathic phenotype. Caveolin-1 KO mice show complete ablation of the presence of the caveolae, cellular organelle, in the endothelium and fat. Similarly, caveolin-3 KO mice lack caveolae in cells that normally express this protein such as skeletal muscle, heart and diaphragm. Heart tissue is made up of different types of cells. Differentiated cardiomyocytes surrounded by a network of cardiac fibroblasts and endothelial cells and less abundant vascular smooth muscle cells. There is also a controversy regarding expression of caveolin isoforms in the heart muscle. It is well known that cardiac myocytes express caveolin-3 and other cell types in the heart express caveolin-1 and caveolin-2. But recent studies provided the evidence of the existence of caveolin-1 in cardiomyocytes [61].

### Caveolin and Atherosclerosis

Experimental evidence indicates that caveolae and caveolins have the possibility to influencing atherogenesis in many ways. Caveolin-1 is a cholesterol-binding protein that can transport cholesterol from the endoplasmic reticulum (ER) to the plasma membrane. The major receptors for high-density lipoprotein, SR-B1, and a scavenger receptor for modified forms of LDL, CD36, can also reside in and signal in caveolae-type microdomains [62]. In addition, oxidized LDL can extract caveolae cholesterol, unlocalize eNOS, and impair NO release [63]. Conversely, blockade of HMG CoA reductase with statin-based drugs reduces caveolin levels and promotes eNOS activation [64]. This concept has been validated in apolipoprotein E-deficient (ApoE^–/–^) mice where statin treatment decreases caveolin-1 expression and promotes NOS function *in vivo* [65]. However, to date, there are no data showing changes in caveolin-1 levels in atherosclerotic lesions from humans [60].

To verify, if caveolin-1 influenced lesion progression in mice, Lisanti and his coworkers crossbred caveolin-1^–/– ^mice with ApoE^–/–^ mice that develop atheromas. Interestingly, the loss of caveolin-1 in the ApoE^–/–^ mice resulted in a proatherogenic lipid profile, similar to that seen in CD36^–/–^ mice bred to an ApoE background [66,  67]. Surprisingly, despite a pro-atherogenic lipid profile, the loss of caveolin-1 reduced lesion burden by 80%, suggesting caveolin-1 regulated LDL-mediated vascular dysfunction, inflammation, and lesion progression. The authors suggested this may be caused by a decrease in stability of the scavenger receptor for oxidized or modified LDL, CD36 in macrophages, and an increase in endothelium-derived NO production, which would reduce vascular inflammation. These remarkable findings unequivocally support the importance of caveolin-1/caveolae in the pathogenesis of atherosclerosis [60].

### Caveolin and Cardiac Hypertrophy

Cardiac hypertrophy is the consequence of an increase in cardiac myocyte size and/or mass. Since cardiac myocytes have no capacity for cellular proliferation, their only means of growth is by cellular enlargement. Given that cardiac failure is the most common result of insufficiency of myocardium, it is not surprising that cardiomyocyte hypertrophy is the dominant cellular response to virtually all forms of hemodynamic overload [68]. However, long-term adaptive/compensatory hypertrophy is associated with progressive ventricular dilation. As a consequence of cardiac enlargement and wall thinning, stress on the wall also increases, despite constant intracavitary pressure. This mathematical increase in wall stress generates its own hemodynamic stress on the heart, further stimulating overloaded hypertrophy signaling pathway and thereby altering the balance from cell growth response to cell death. Once these processes have progressed to this stage (decompensation, loss of cardiac myocytes), irreversible functional deterioration develops, which leads to heart failure and, ultimately, death [69, 70]. 

Overexpression of caveolin-3 in neonatal cardiac myocytes decreases the ability of the adrenergic agonist phenylephrine or endothelin-1 to increase cell size [71]. A similar kind of effect is seen in cardiac myoblasts (H9C2) in which cav-3 reduces angiotensin II–promoted hypertrophy [72]. Other studies indicate that cardiac hypertrophy results in decreased expression of cav-3 [73, 74] and that right heart [73] left heart [75] hypertrophy is enhanced in caveolin-1 KO and caveolin-1/3 double KO mice. Down regulation of growth signals are the most likely cause of expressed caveolin induced inhibition of cardiomyocyte gowth. Cav-1 and -3 KO mice show hyperactivation of p42/44 MAPK [76] and upregulation of eNOS activity and nitrosative stress [74, 75, 61]. By contrast, increased caveolin expression down regulates activity of those entities [71, 77]. Chronic myocardial hypoxia increases eNOS expression while decreasing the expression of cav-3, consistent with the idea that the expression and activity of eNOS is dependent on caveolin [78]. Alterations in caveolin expression almost certainly change the ability of the hypertrophied heart to respond to a variety of physiologic and pharmacologic agonists/ stimulus [61].

### Caveolin and Myocardial Ischemia

Ischemic heart disease is major problem in Western society and a major cause of death and disability. Precondition (PC) is the phenomenon whereby brief episodes of ischemia and reperfusion render the heart resistant to ischemic injury from a subsequent ischemic insult. Thus, ischemic PC is a protective and adaptive mechanism produced by short periods of ischemic stress rendering the heart more protected against another similar or greater stress. Early preconditioning depends on adenosine, opioids and to a lesser degree, on bradykinin and prostaglandins, released during ischemia. This molecule activate G-protein coupled receptor, initiates activation of K_ATP_ channel and generate oxygen free radicals, and stimulate a series of protein kinases, which include protein kinase C, tyrosine kinase and members of MAP kinase family. Late preconditioning is triggered by a similar sequence of events, but in addition essentially depends on newly synthesized proteins, which comprise iNOS, COX-2, manganese superoxide dismutase and possibly heat shock proteins. The final mechanism of PC is still not very clear. However, evidence is rapidly accumulating about the involvement of caveolin or caveolae in cardioprotection against myocardial ischemia and ischemia/reperfusion injury [79].

**Fig. (1):**
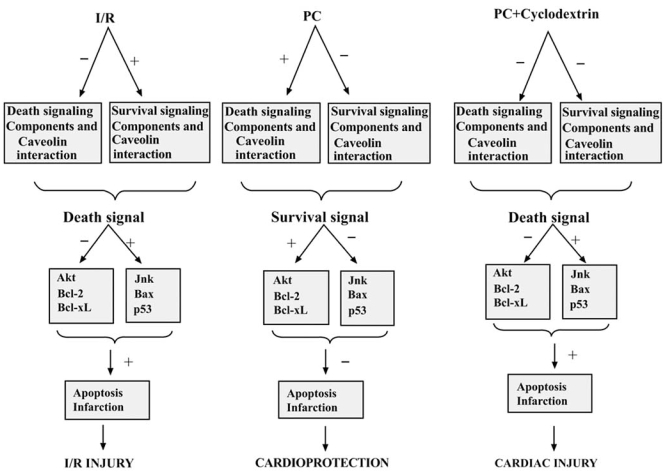
Proposed model of the role of lipid raft in the ischemic preconditioning of the heart. In I/R heart, anti-death signaling components (p38MAPKβ and ERK 1/2) remain bound (+) with caveolin, whereas there was reduced association (-) of death signaling components (p38MAPKα, JNK and caspase-3) with caveolin. These unbound death signaling components induces reperfusion injury in the heart by expressing (+) JNK, BAX and p53 in the myocardium. In PC heart, death signaling components remain bound (+) with caveolin, whereas there was reduced association (-) of anti-death signaling components with caveolin. These unbound anti-death/survival signaling components induced cardioprotection by expressing (+) AKT, Bcl-2 and Bcl-xl in the myocardium. When precondition was performed in presence of cyclodextrin, lipid raft disintegrator, there was no particular strong interaction of survival signaling components or death signaling components with caveolin. Due to the loss of fine control on the availability death and survival signals, heart can not generate survival signal (cardioprotection) in the PC heart in presence of lipid raft disintegrator, which was further confirmed by the expression (+) of JNK, BAX and p53 in myocardium of cyclodextrin treated heart. [I/R= ischemia reperfusion, PC= precondition]. [Reproduced from Fig. (**8**) of Cell Physiol Biochem 2008; 21: 325-334 with permission from Karger].

Ischemia/reperfusion injury activates p42/44 and p38MAPK, redistributes caveolin-3 and downregulates expression of caveolin-1 [80]. Disruption of caveolae using MβCD eliminates the ability of ischemia and pharmacological preconditioning to protect the cardiac myocyte from injury [81]. This is also supported by the decreased ability of Cav-1 KO mice to undergo pharmacological preconditioning [82]. Recent investigations also showed that pro-survival signaling components (e.g., ERK1/ 2, HO-1, eNOS and p38MAPKβ) translocate and/or interact with caveolin in ischemia/reperfusion heart and render the heart less abundance to pro-survival signal and induces myocardial injury. Similarly, in preconditioned heart death signaling components (e.g., p38MAPKα, JNK and Src) translocates and/or interact with caveolin in preconditioned heart and rendering the heart less exposed to death signaling components and more abundant to pro-survival signaling components [83, 84]. Although detail mechanism of action of caveolin is not very clear, but evidence indicates that proteasomes play a very important role in the interaction between caveolin and signaling components. However, overall observation indicates that caveolin plays a pivotal role in cardioprotection against ischemic injury (Fig. **1**).

## CONCLUSION

Caveolae and caveolins are undoubtedly regulating various aspects of cardiovascular system. Clearly loss of caveolin-1 has profound effect on the eNOS pathway, indicating the importance of this interaction, whereas the loss of caveolin-3 impacts NOS as well as MAPK activation. Although detail mechanisms of actions are not very clear, experimental evidences demonstrate the predominant role of caveolin in cardiac hypertrophy, atherosclerosis, ischemic injury and different myocardial functions. Recent investigations are disentangling the complex processes of caveolin regulated signaling systems in the myocardium and developing novel approaches, aimed at counteracting cardiomyocyte apoptosis in heart failure and/or cardiovascular diseases.
